# Atomically Precise Detection and Manipulation of Nitrogen-Vacancy Centers in Nanodiamonds

**DOI:** 10.1021/acsnano.2c10122

**Published:** 2023-04-07

**Authors:** Bethany M. Hudak, Rhonda M. Stroud

**Affiliations:** Materials Science and Technology Division, U.S. Naval Research Laboratory, Washington, D.C. 20375, United States

**Keywords:** nanodiamond, single photon emitters, quantum materials, color centers, atomic resolution

## Abstract

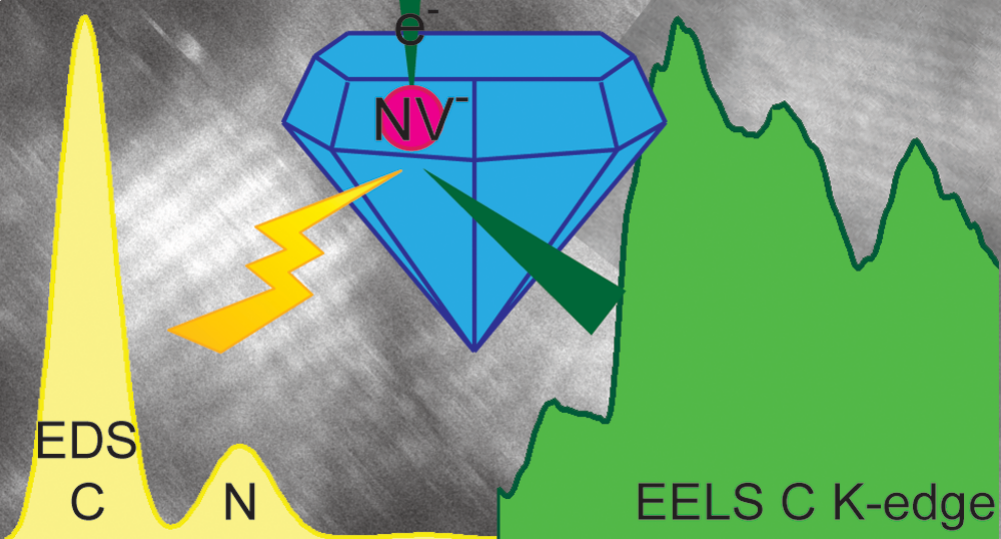

Nitrogen-vacancy (NV) centers in nanodiamonds are a promising quantum communication system offering robust and discrete single photon emission, but a more thorough understanding of properties of the NV centers is critical for real world implementation in functional devices. The first step to understanding how factors such as surface, depth, and charge state affect NV center properties is to directly characterize these defects on the atomic scale. Here we use Angstrom-resolution scanning transmission electron microscopy (STEM) to identify a single NV center in a ∼4 nm natural nanodiamond through simultaneous acquisition of electron energy loss and energy dispersive X-ray spectra, which provide a characteristic NV center peak and a nitrogen peak, respectively. In addition, we identify NV centers in larger, ∼15 nm synthetic nanodiamonds, although without the single-defect resolution afforded by the lower background of the smaller natural nanodiamonds. We have further demonstrated the potential to directly position these technologically relevant defects at the atomic scale using the scanning electron beam to “herd” NV centers and nitrogen atoms across their host nanodiamonds.

Diamonds are an ideal system for scalable quantum technology, and are also mechanically hard, chemically inert, and optically transparent.^1,2^ Nitrogen-vacancy (NV) and silicon-vacancy (SiV) centers in diamonds, where a substitutional dopant sits adjacent to a lattice vacancy with a trapped electron, can be engineered as single photon emitters (SPE) for use in applications in quantum communication and bioimaging.^3^ In particular, NV color centers are room temperature SPEs with a coherence time of longer than one second and are thus promising for quantum information technologies.^4−7^ However, the properties of NV centers in diamonds can be impacted by characteristics such as charge state and nanodiamond surface,^8^ and it is therefore crucial to develop a more complete atomistic understanding of the system.^9^

Quantum-scale properties are difficult to probe, and such studies are limited to a few techniques. Scanning transmission electron microscopy (STEM) enables routine atomic-resolution imaging and spectroscopy in many systems, but single-atom or defect sensitivity is not universally accessible. Nitrogen was first observed in diamond platelets using electron energy loss spectroscopy (EELS) in STEM.^10^ Point defects are more challenging to locate and characterize than one- or two-dimensional defects, but low-loss STEM-EELS is theoretically capable of resolving an NV center in diamonds to within 1 nm.^11^ In this study, we use simultaneous core-loss STEM-EELS and energy dispersive X-ray spectroscopy (EDS) to confirm the presence of NV centers in natural meteoritic and synthetic nanodiamonds. Natural nanodiamonds with astrophysical origins are a good test system for studying NV centers in diamonds on an atomic scale because they are known to contain ∼ 0.1 at. % N, and their small size (<5 nm) means they are likely to contain only one NV center. Recent calculations have shown that the NV center in diamonds produces a core-loss EELS peak at 282.4 eV preceding the C K-edge, with greater spatial localization than low-loss features.^12^ By concurrent acquisitions of core-loss EELS and EDS spectrum images (SI), we can confirm that the edge peak indeed arises from the NV center in cases where the 282.4 eV peak in EELS coincides with the 0.392 keV N peak in EDS.

## Results and Discussion

There are a number of proposed potential origins of the 282.4 eV peak,^13,14^ and the recent suggestion that it is the result of the NV center requires an independent confirmation, beyond just detection of the pre-edge peak in nanodiamond. Due to the similar atomic numbers of N and C, N impurities in the nanodiamond lattice cannot be directly observed in Z-contrast imaging. Further, the EELS edge of a single N atom in a nanodiamond is difficult to distinguish from background, because the signal at the N K-edge in diamonds is dominated by the tail of the C K-edge. However, EDS is sensitive enough for single-atom spectroscopy.^15,16^ Stroud et al. identified a single Si atom on amorphous carbon in under 10 s.^16^ Because the relative k-factor of N:Si is 1.174, under similar imaging conditions, EDS detection of a single N atom in nanodiamond should be possible. Given that the NV center produces both a 282.4 eV peak in EELS and the 0.392 keV N peak in EDS, we can further distinguish between NV centers and other N species that may be present, such as surface or interstitial N, by locating N EDS peaks in the absence of the 282.4 eV EELS feature.

**Figure 1:**
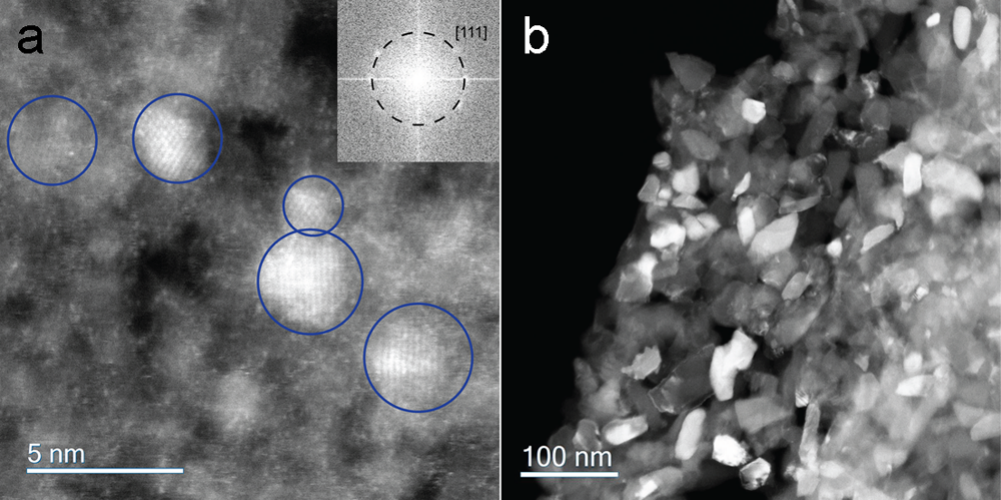
STEM-MAADF images of meteoritic and synthetic nanodiamonds showing average size and distribution. (a) Nanodiamonds from the Murchison meteorite embedded in an amorphous carbon matrix. Blue circles highlight several small nanodiamonds. (Inset) FFT with (111) plane of diamond indexed. (b) Synthetic nanodiamonds with average diameter of 30 nm.

**Figure 2:**
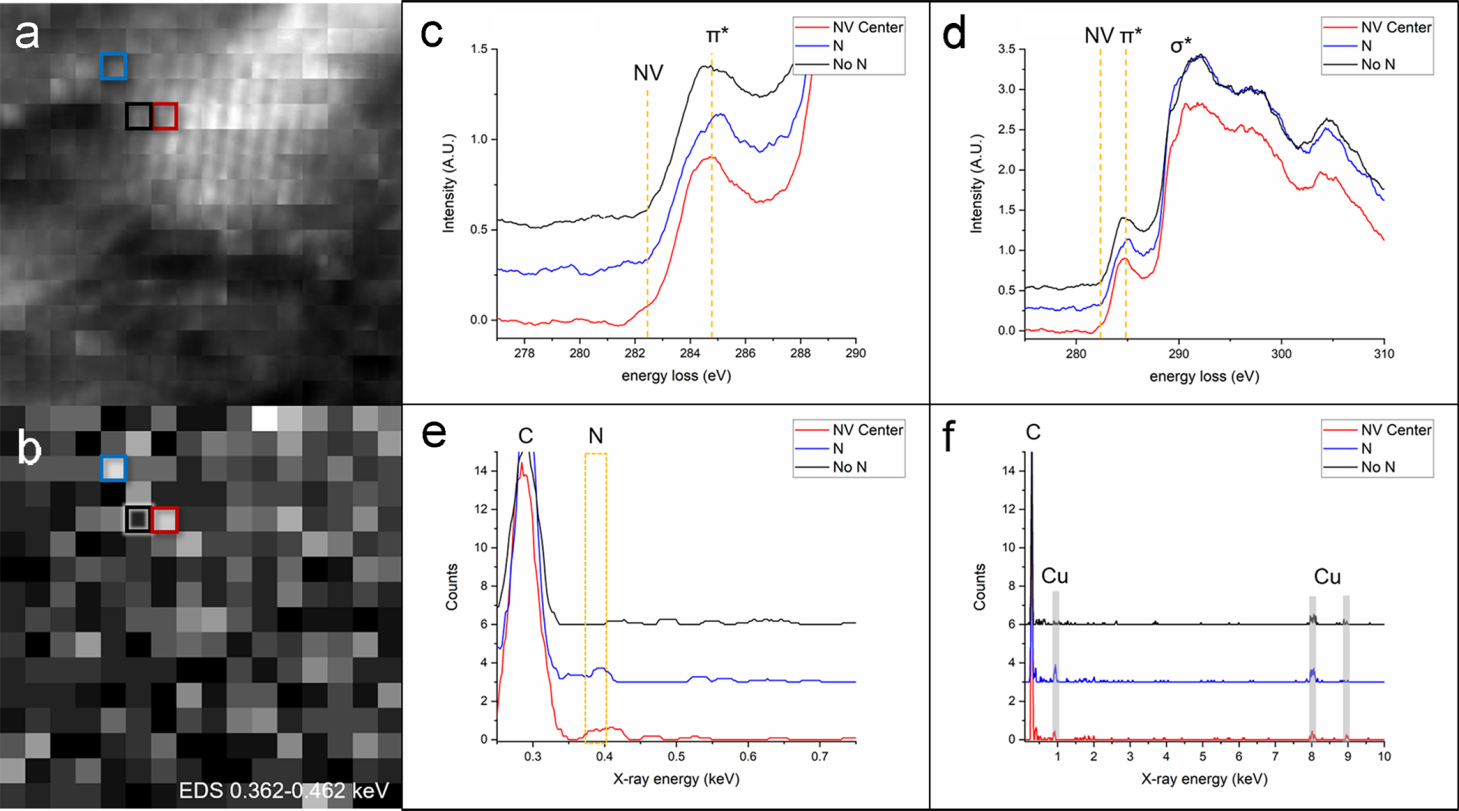
Simultaneous STEM-EELS/EDS spectrum imaging of a meteoritic nanodiamond. (a) HAADF image acquired during SI. (b) EDS SI with intensity averaged from 0.362–0.462 keV. (c) Portion of the EELS spectra smoothed by 35 point adjacent-pixel averaging and cropped to visualize the spectra at 282.4 eV. (d) Full view of the EELS C K-edge displaying characteristic diamond features. (e) Portion of EDS spectra smoothed by 8 point adjacent-pixel averaging displaying C and N peaks. (f) Full EDS spectra with C and Cu system peaks visible. (c–f) Red, black, and blue curves correspond to the red, black, and blue pixels selected in the SI (a,b).

For this study we chose to investigate NV centers in meteoritic nanodiamonds isolated from the Murchison meteorite. These are a good test system because the nanodiamonds average 2–5 nm in diameter, with a N:C ratio of ∼ 1:1000, limiting the nanodiamond, on average, to only a single NV center.^17^ The small particle size also provides lower background in both EELS and EDS data, and reduces the probability of N X-ray photon reabsorption in the sample for greatest N EDS sensitivity. Figure 1a is a representative STEM medium-angle annular dark-field (MAADF) image displaying several small nanodiamonds (circled in blue) embedded in amorphous carbon, with Figure 1b displaying a MAADF image of synthetic nanodiamonds. The Figure 1a inset FFT indexes to the (111) plane of diamond. Twenty-six STEM-EELS/EDS SIs from meteoritic nanodiamonds with adequate signal-to-noise ratio (SNR) and spatial resolution were analyzed. Each SI contained 225–400 pixels, with each pixel containing high-angle annular dark-field (HAADF) contrast, an EEL spectrum, and an EDS spectrum. Each pixel was analyzed to determine if it contained an EELS peak at 282.4 eV and/or an EDS peak at 0.392 keV. Figure 2 displays the best example of an NV center in a nanodiamond. Figures S1–S2 in the Supporting Information show two additional NV centers identified using STEM-EELS/EDS. Figure 2a displays the HAADF image acquired with the corresponding EDS SI, displaying intensity from 0.362 to 0.462 keV, shown in Figure 2b. In each panel, three colored boxes indicate pixels that contain an NV center (red), a N without a neighboring vacancy (blue), and a control pixel containing neither feature (black). Figure 2c,d displays the EELS data corresponding to each colored box, with Figure 2c containing a zoomed in region of the spectra, normalized to the π* peak at 285 eV, to highlight the 282.4 eV feature originating from the red pixel. Figure 2d shows a wider view of the C K-edge with a clear σ* peak, confirming that the particle is indeed diamond. Figure 2e,f displays the simultaneously acquired, corresponding EDS data. Figure 2e is focused on the portion of the spectra containing N at 0.392 keV. The C peak is also visible at 0.277 keV. A wider view of the EDS spectra is shown in Figure 2f, where the Cu peaks from the microscope pole piece cover are present at 0.923 and 8.041 keV. EDS simulations using NIST DTSA-II software were performed to qualitatively compare to the experimental data (Figure S3). The simulated EDS show N signal above background at as low as 1 at. % N in diamonds. The red box in Figure 2a,b highlights a single pixel in the SI containing both the EELS peak at 282.4 eV and the N EDS peak at 0.392 keV. The EELS feature occurs concurrently with the presence of N, and we can thus conclude that this pixel contains a single NV center. The adjacent pixel (Figure 2a,b black box), for comparison, shows neither the 282.4 eV peak in EELS nor the N peak in EDS. The blue box in Figure 2a,b contains the N EDS signal but no 282.4 eV peak in EELS has also been identified, which we conclude is a surface or interstitial N atom, as it does not produce the EELS peak characteristic of an NV center.

Long acquisition times were required to generate enough signal from a single atomic defect, but at the same time too much exposure would damage the sample. Eight seconds per pixel, resulting in full SIs that took upward of 90 min to acquire, was determined to be sufficiently long enough to generate a signal without damaging the <5 nm nanodiamonds. Through this approach, enough signal was generated to experimentally verify the computational result from Chang et al.:^12^ that the 282.4 eV EELS peak originates from an NV center in diamonds.

**Figure 3:**
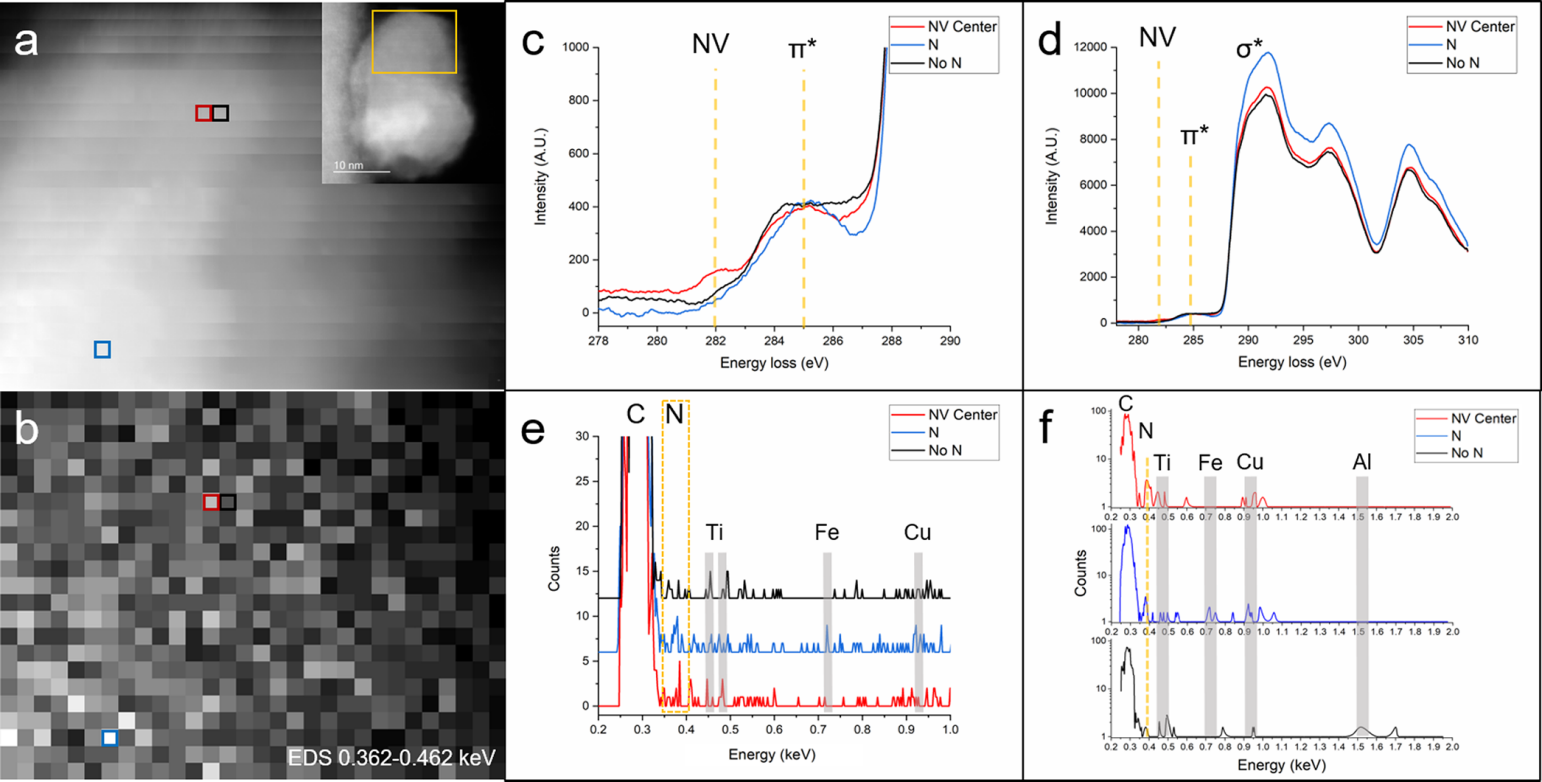
Simultaneous STEM-EELS/EDS spectrum imaging of a synthetic nanodiamond. (a) HAADF image acquired during SI. Inset: Entire nanodiamond with yellow box indicating the region from which the SI was acquired. (b) EDS SI with intensity averaged from 0.362–0.462 keV. (c) Portion of the EELS spectra smoothed by 35 point adjacent-pixel averaging and cropped to visualize the spectra at 282.4 eV. (d) Full view of the EELS C K-edge displaying characteristic diamond features. (e) Portion of EDS spectra displaying C and N peaks as well as Ti, Fe, and Cu. (f) EDS spectra plotted on a log scale to show the N peaks above background and artifact peaks. (c–f) Red, black, and blue curves correspond to the red, black, and blue pixels selected in the SI (a,b).

Meteoritic nanodiamonds are complicated by poorly constrained formation and processing histories, and their small size and surface oxidation makes them susceptible to amorphization under long beam exposures. To expand our study to nanodiamonds with well-constrained growth history, which can be mass produced with NV centers for scalable quantum technology, we repeated these studies on synthetic nanodiamonds. The nanodiamonds used here were synthesized from 1b powders containing N impurities^18^ that were irradiated with a 40 keV He+ beam to create the negatively charged NV centers.^19^ A representative image is shown in Figure 1b. The nanodiamonds average 30 nm in diameter, small enough for single-atom EDS detection.^15^ By acquiring EELS and EDS SIs using the same parameters as with meteoritic nanodiamonds, we can again identify pixels that contain an NV center, no N, and N that does not form an NV center. Figure 3a shows the HAADF image acquired during spectrum imaging. Inset in Figure 3a is a MAADF image of the full nanodiamond, approximately 20 nm wide by 30 nm tall, with a yellow box indicating the region of the SI. The red, black, and blue boxes in Figure 3a,b highlight pixels that contain an NV center, no presence of N, and N without a coordinated vacancy, respectively. Figure 3b is the EDS SI displaying the intensity from 0.362 to 0.462 keV. Figure 3c,d contains the EEL spectra from the three pixels and has been normalized to the π* peak. Figure 3e,f shows the corresponding EDS spectra. As with the meteoritic data set, the red pixel contains an EELS peak at 282.4 eV, indicative of an NV center, and an N peak at 0.392 keV in the EDS. As a control, the adjacent black pixel is representative of diamond and contains neither signal. The blue pixel contains N, as evidenced by EDS, but has no 282.4 eV feature in EELS, indicating that it is not coupled to a vacancy. These nanodiamonds contain much less sp^2^ carbon than the meteoritic nanodiamonds, making the 282.4 eV peak easier to distinguish. The extended EEL spectra (Figure 3d) are characteristic of diamond, and the EDS spectra (Figure 3f) show C, N, Fe, Ti, Al, and Cu. The synthetic nanodiamonds contained more artifact peaks than the meteoritic nanodiamonds. The thicker synthetic diamonds produce more backscattering, which leads to system peaks, like Cu, Fe, and Ti. Additional peaks are impurities resulting from manufacturing conditions. Figure S4 shows an EDS spectrum acquired over a 512 nm × 512 nm region of nanodiamonds with multiple artifact peaks labeled.

The N EDS signal provides an important confirmation of the assignment of the 282.4 eV feature to NV centers and can help in quantification of the total number of NV centers detected. Prior experiments demonstrated that under the measurement conditions used here, a single Si atom on the 2 nm nanodiamond surface can be detected with EDS in less than 10 s.^16^ Given N’s higher k-factor than Si, single N detection is also possible given our imaging conditions, as long as the samples are thin enough for absorption of the N X-ray by the C sample to be negligible. Because the meteoritic nanodiamonds are 2 to 4 nm, with an overall N concentration such that only one NV center should appear in a 0.2 nm probe spot, we are confident that we are indeed detecting single defects, opposed to multiple defects in a single atomic column. Other efforts have been made to measure NV centers in diamonds through the use of low-loss STEM-EELS, but this approach requires a monochromated microscope and a nearly pristine sample surface.^20^

**Figure 4:**
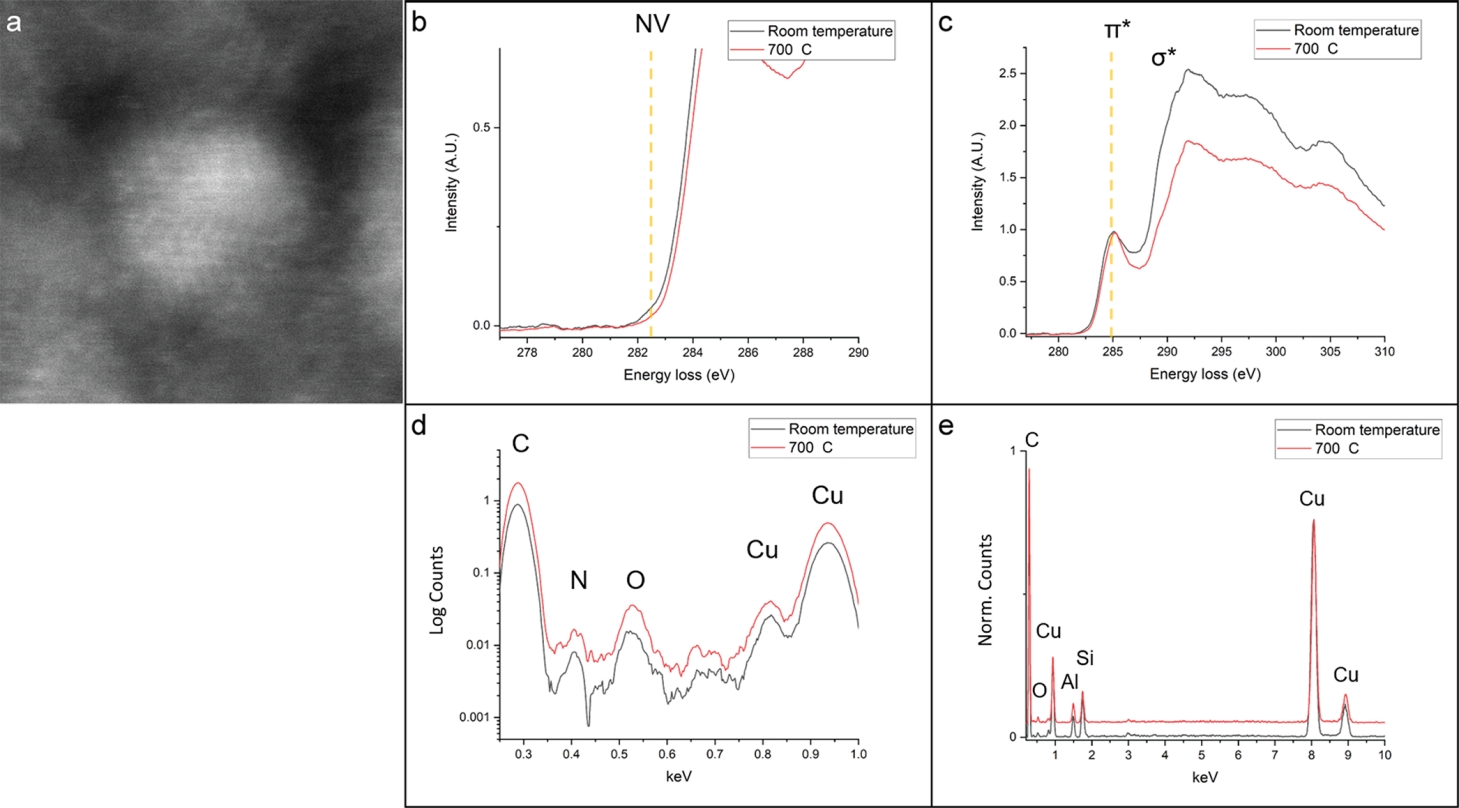
NV centers in meteoritic nanodiamonds at elevated temperature. (a) MAADF image of a nanodiamond at room temperature. (b) Portion of the EEL spectra smoothed by 10 point adjacent-pixel averaging, normalized to the π* peak at 285 eV and cropped to show the signal at 282.4 eV. There is a decrease in signal intensity from room temperature (black curve) to 700 °C (red curve). (c) Full EEL spectra at room temperature (black) and after heating to 700 °C (red) normalized to the π* peak at 285 eV with a σ* peak indicative of diamond. (d) EDS spectra at room temperature (black) and after heating to 700 °C (red) plotted on a log scale and zoomed in to show N intensity. There is a decrease in N and O after heating to 700 °C in UHV. (e) Full EDS spectra at room temperature (black) and after heating to 700 °C (red) normalized to the C peak. Cu, Al, and Si artifact peaks are visible.

After confirming the capability to detect a single NV center, we investigated the reaction to applied heat. The meteoritic nanodiamond residue was dropcast onto a Protochips thermal chip for *in situ* heating. Due to the geometry of the sample cartridge and the heating chip, greater collection time is needed to acquire an N EDS signal above background. Therefore, instead of acquiring SI, reduced-area summed spectra were taken. This approach also limited the ability to see the peak at 282.4 eV because the spectrum is averaged over several frames. A MAADF image of the nanodiamond before heating is shown in Figure 4a. At room temperature, the nanodiamond shows a small feature in the EEL spectrum at 282.4 eV (Figure 4b)—cropped from the full EELS acquisition (Figure 4c)—and a small N peak in the EDS spectrum (Figure 4d), indicating the presence of at least one NV center. For clarity, EDS data in Figure 4d is displayed on a log scale. The sample was then heated to 700 °C in the TEM column under ultrahigh vacuum, where it dwelled for 1 h before ramping back to room temperature. The thermal glow of the heating chip prevented N from being imaged at temperature by EDS. The photons from the chip broaden the strobe peak at 0 keV, obscuring the 0.392 keV N peak. Simultaneous EELS and EDS data were acquired from the same nanodiamond after heating. The peak at 282.4 eV diminishes (Figure 4b), and in EDS, the N peak decreases but does not completely disappear (Figure 4d). We expect some N to be released from the nanodiamond above 500 °C^17^ and any N-bearing organic contamination on the surface to have sublimed at elevated temperature. The area under the N peak was integrated before and after heating, yielding 0.21 and 0.15 counts per second, respectively, and in both cases a 2-fold increase over the background intensity. The dissipation of the EELS peak at 282.4 eV indicates that the NV center defect is modified in some manner, with one possibility being that the defect is passivated by H from adjacent amorphous organic carbon. The EDS data also shows a prominent O peak from surface contaminants. Upon heating, this peak also decreases. It is expected that organic surface contaminants sublimate from the surface into the TEM vacuum at these temperatures. Due to the geometry of the heating cartridge, there are many more artifact peaks in the EDS spectra (Figure 4e), with Cu, Al, and Si being present. Further research is necessary to fully understand the behavior of the nanodiamond NV center at elevated temperatures.

In addition to being able to unambiguously locate single-atom qubits, scalable quantum technology requires precise fabrication of these systems. It has recently been demonstrated that the STEM can be used for atomic-scale material fabrication and modification.^21−24^ The primary energy used for imaging these nanodiamonds is 60 kV. At this accelerating voltage, the maximum kinetic energy transferred from the beam to a N in a single elastic collision along the beam direction is 9.95 eV.^25^ The migration energy barrier of an NV center and a substitutional N in diamonds is 4.9 and 5.0 eV, respectively, well below the energy imparted from the electron beam.^26,27^ Because the beam position in STEM can be manually controlled, it is therefore possible that the atomically-precise electron beam can be used to guide the placement of N atoms and/or NV centers in diamonds.

**Figure 5:**
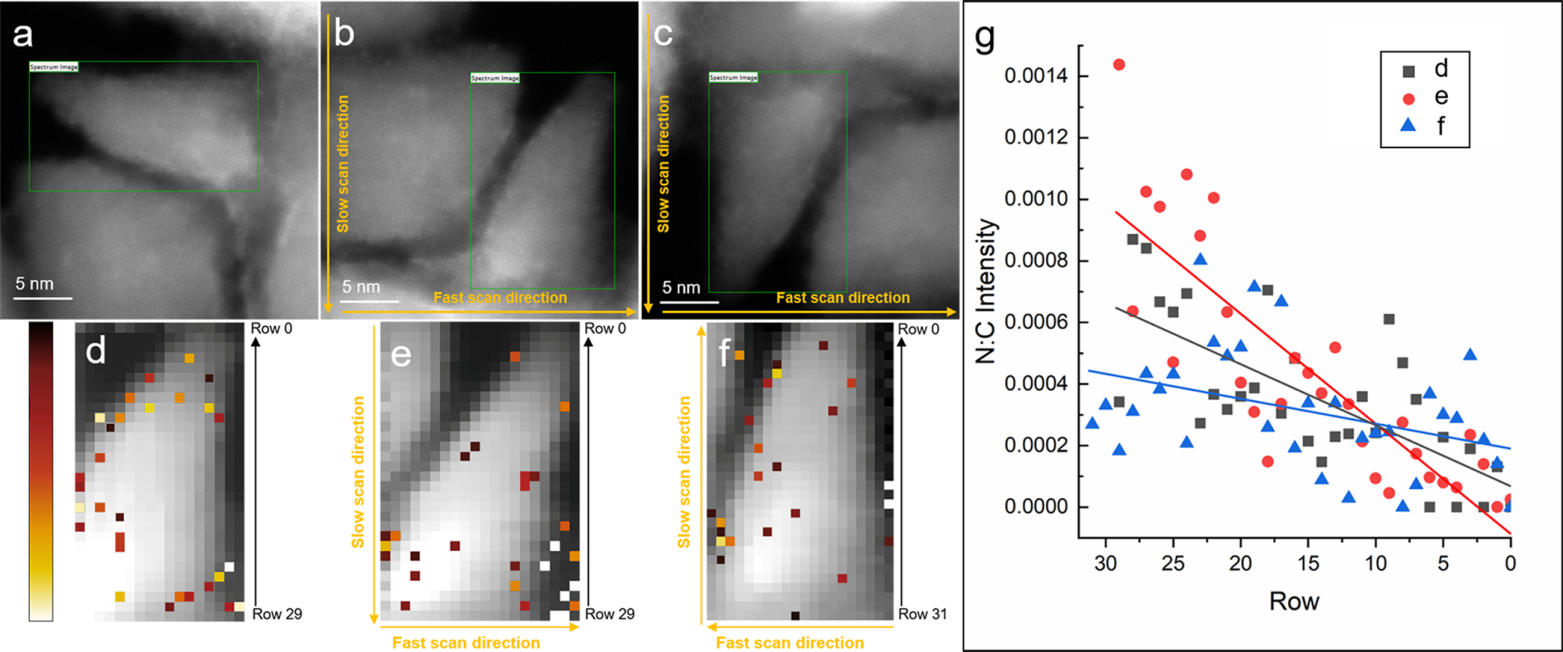
Electron-beam guided migration of N atoms. (a–c) HAADF image of a nanodiamond rotated (b) 90° and (c) 180°. (b) The beam was scanned over the nanodiamond at a rate of 16 μs/pix for 87 min. (c) The beam was scanned over the nanodiamond at a rate of 24 μs/pix for 180 min. (d) N:C EDS intensity overlaid on EELS SI of the nanodiamond. (e) N:C EDS intensity overlaid on EELS SI of the nanodiamond after the 87 min scanning period. (f) N:C EDS intensity overlaid on EELS SI of the nanodiamond after the 180 min scanning period. (g) Scatter plots of EDS N:C intensity averaged across each row in d–f.

To determine if the electron beam can be used to move the N and/or NV centers in the nanodiamonds, we performed repeated scans over a single synthetic nanodiamond, then used EELS and EDS SI to track the position of N atoms. The nanodiamond in Figure 5a was imaged and spectroscopically mapped with a beam current of approximately 180 pA. The EDS SIs were used to determine the position of N atoms at three points in time. First, an SI was acquired from the nanodiamond (Figure 5a). Second, the image was rotated 90°, and the beam scanned over the nanodiamond at 16 μs/pix for 87 min (Figure 5b). Third, the image was rotated 180°, and the beam was scanned at a rate of 24 μs/pix for 180 min (Figure 5c). Simultaneous EELS and EDS SIs were acquired after each extended scanning period. To visualize the position of N atoms, the EDS SI was sliced at 0.392 ± 0.0075 keV to create a N map and 0.2857 ± 0.0437 keV to create a C map. A simple background subtraction was performed on each map, and the N map was divided by the C map. This produced a map of N normalized to the C intensity, which is overlaid on the C EELS spectra to show the position of N in relation to the nanodiamond (Figures 5d–f). The contrast limits were adjusted to highlight the position of N atoms. After the 87 min scanning period, there is an increase in N:C concentration in the slow-scan direction of the image (Figure 5e), i.e., the N atoms have migrated in the direction in which the beam was scanned. The bottom-right region of Figure 5e has the highest concentration of N. After rotating and scanning over the image a second time, the EDS SI (Figure 5f) now shows the N redistributed into the nanodiamond, successfully demonstrating that the focused electron beam can be used to move N atoms in a nanodiamond. Figure 5g displays three scatter plots of the average N:C intensity across each row, with a linear fit for each. Row 0 is the narrowest portion of nanodiamond, indicated in Figures 5d−f. In each case, there is a decrease in N from the widest to the narrowest part of the nanodiamond. However, the second plot (after the 87 min scanning period) shows a larger average N ratio at the widest portion of the nanodiamond—the direction in which the N were being herded by the beam—than the other two plots. The third plot (after the 180 min scanning period) shows a flatter slope, suggesting a more even distribution of N throughout the nanodiamond. This is a strong indication that the N is indeed migrating in the slow-scan direction of the electron beam.

**Figure 6:**
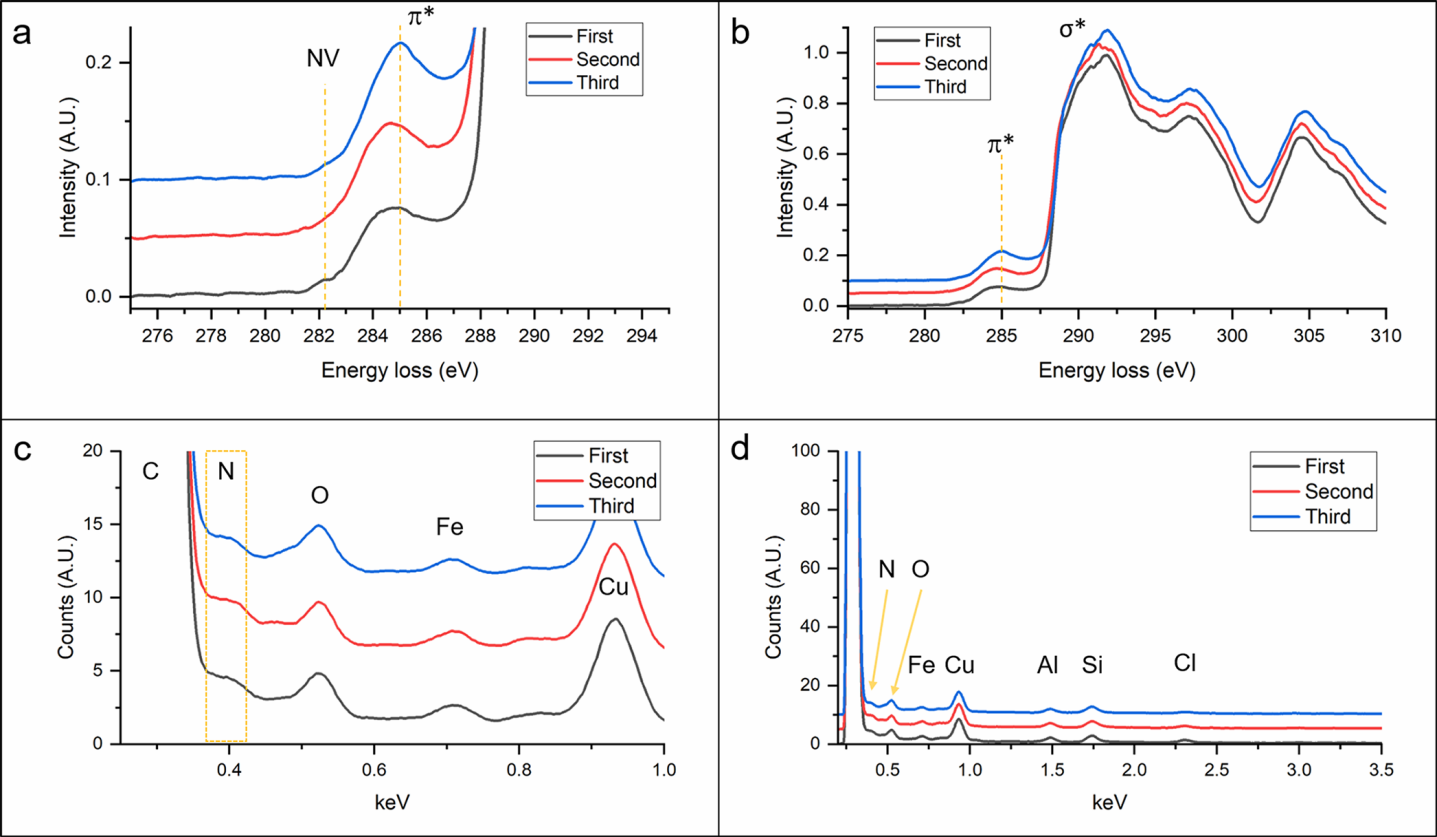
EDS and EELS summed spectra data from the three scanning steps described in Figure 5. (a) EEL spectra summed over entire SI normalized to the σ* peak at 290 eV and zoomed in to see the NV peak at 282.4 eV. The data have been smoothed by 35 point adjacent-pixel averaging in Origin Pro 2022. (b) Full EEL spectra summed over the entire SI. (c) EDS data zoomed in to see the N peak. (d) Wide view of EDS data with artifact peaks of O, Fe, Cu, Al, Si, and Cl.

Figure 6 shows the EEL spectra summed over the entire SI to determine the impact N migration has on NV centers. Surprisingly, the EELS peak at 282.4 eV decreases after the 87 min scanning period, as evident in Figure 6a, which has been cropped from the full spectrum in Figure 6b. This could indicate that the N are clustering to form multiple-N structures or N platelets. This is consistent with literature studying N diffusion in diamonds, which predicts that mobile N can be trapped by interstitial N to form N aggregates. When the N is diffused back into the nanodiamond after the 180 min scanning period, the peak at 282.4 eV returns, indicating that the N aggregates have dissociated and the isolated N have been able to recombine with lattice vacancies to form NV centers. The EDS spectra in Figure 6c,d show that the N peak remains unchanged, suggesting that the total quantity of N does not vary significantly.

This work demonstrates that the STEM beam can be used to reposition NV centers in diamonds; however, questions remain as to the exact mechanism of NV center movement. The field of directly positioning atomic defects in 3D materials using a STEM probe is still developing, and one lesson learned from previous work is that the mechanism of defect positioning can be surprising.^28^ Further experimental studies on atomically resolved diamond crystals using point-by-point defect positioning, similar to the approach of previous atomic manipulation studies,^23,28−30^ will provide additional insight into how these defects move through the lattice. Molecular dynamics (MD) and density functional theory (DFT) are also necessary to ascertain the true mechanism of NV center displacement. We can, however, speculate about the mechanism using diamond literature studying the diffusion of N and NV centers as well as knowledge of beam–sample interactions.

**Figure 7:**
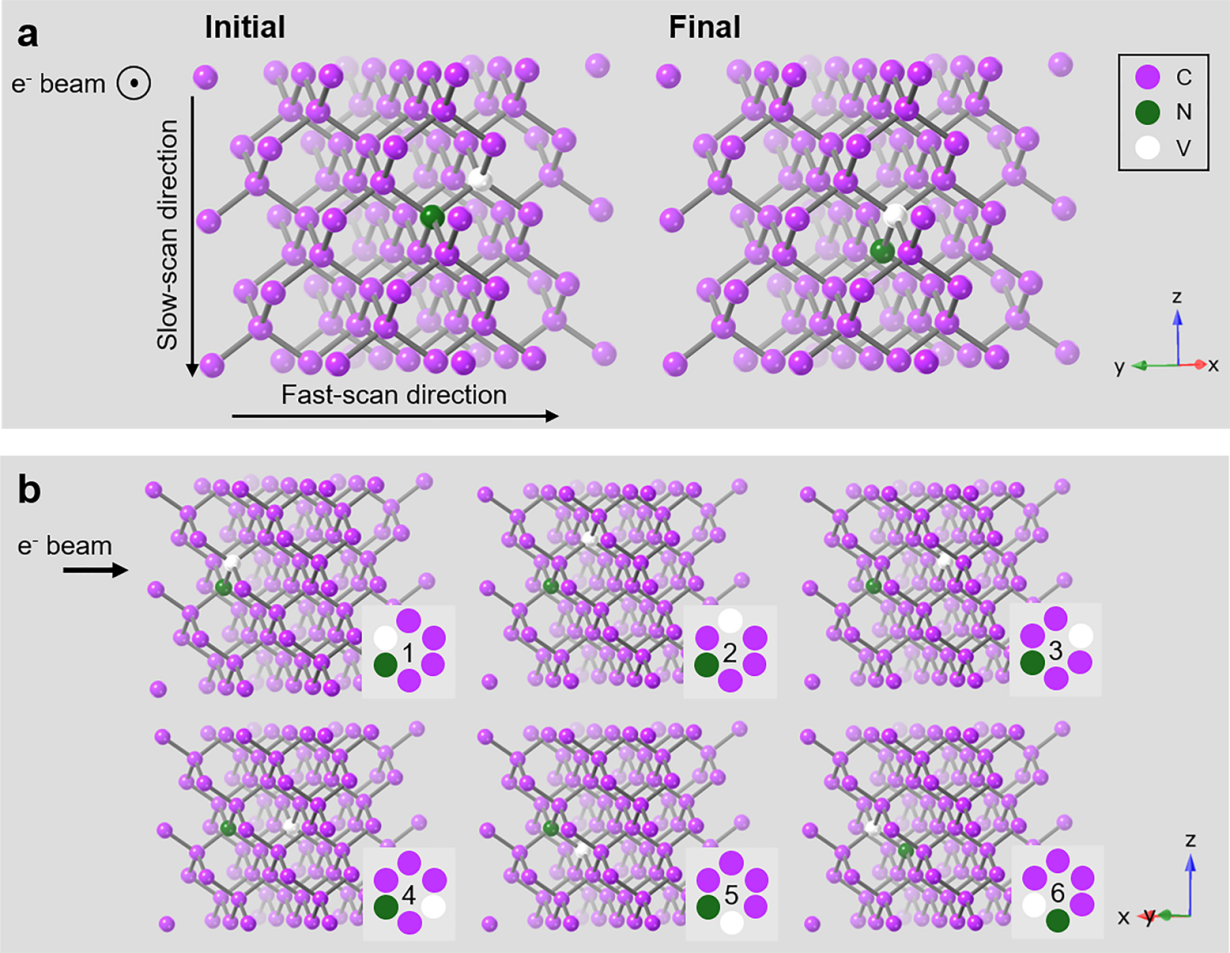
Proposed mechanism of NV center diffusion in a diamond lattice. (a) Initial and final position of an NV center viewed along the electron beam axis. The N atom migrates in the slow-scan direction. (b) Diamond lattice rotated 90° about the *Z*-axis with respect to the crystal in panel (a). As the beam strikes the lattice, the vacancy is displaced along the beam axis. It then follows a path along the crystal to recombine with the N atoms and reform the NV center.^31^ Recombination results in the N being displaced by one lattice site. Insets provide 2D representations of the position of the N and vacancy as the vacancy diffuses through the lattice.

Previous work suggests that NV center diffusion is vacancy-mediated, with the vacancy receiving enough energy to diffuse away from the N by a couple lattice sites, but ultimately recombining with N, resulting in displacement of the N.^31^ Unlike in the microscope, those studies introduce energy into the diamond as heat, creating a uniform distribution of energy and equal probability of NV center migration along any lattice direction. In the STEM, however, kinetic energy and momentum from the incident electron is transferred to a local spot approximately the diameter of an atom. Due to the momentum imparted by the electron beam, we propose that the vacancy will diffuse along the beam-axis, similar to the behavior of dopant atoms in thin silicon films.^28^ Because of the brief “kick” from the beam, it is unlikely that the vacancy completely dissociates from the N, in which case the vacancy diffuses through the lattice, and upon recombination, will displace the N by one lattice site. Recombination of the two defects occurs to reduce strain on the diamond lattice imposed by the substitutional N.^32,33^Figure 7 builds upon Pinto et al.’s model of NV center diffusion to illustrate one possible mechanism of N diffusion using the electron beam.^31^Figure 7a displays the initial and final positions of an NV center, as viewed down the beam axis, after electron beam irradiation. Figure 7b is rotated 90° about the *Z*-axis with respect to Figure 7a to show the concurrent steps of vacancy displacement and N recombination if a vacancy is displaced along the beam axis. The total migration barrier is 4.81 eV, below the 11.60 eV transferred to C in a single elastic collision at 60 kV.^25,31^ This is one possible mechanism of NV center diffusion in the slow-scan direction of the electron beam, and further studies are required to verify this hypothesis.

## Conclusions

Nitrogen-vacancy centers in nanodiamonds are promising SPEs with the potential to be integrated into secure quantum communication devices. Here we demonstrate direct detection of a single NV center defect in a nanodiamond using simultaneous STEM-EELS/EDS. The characteristic NV center peak in EELS combined with the N signal in EDS and atomic-resolution imaging provides unambiguous evidence of a single NV center in small 2–5 nm diameter nanodiamonds. NV centers can also be located in larger, synthetic nanodiamonds, but due to the larger background signal in EDS it is likely that multiple N atoms need to be in a single atomic column for detection above background. By repeatedly scanning the beam over a single nanodiamond for long periods of time (1.5–3 h), we are able to “herd” NV centers and/or N atoms in the beam’s slow-scan direction. While further work is necessary to determine the mechanism of beam-guided NV center diffusion, this is an important first step toward individually positioning NV centers to create atomically precise SPE arrays.

## Methods

HAADF and MAADF STEM imaging was performed in an aberration-corrected Nion UltraSTEM 200 equipped with a Gatan Enfinium ER spectrometer for EELS and a 0.7 sr Bruker XFlash windowless SDD-EDS detector. The microscope was operated at 60 kV with the probe current 100–180 pA. Aliquots of samples were dropcast on lacey-carbon-coated copper TEM grids. Simultaneous EELS and EDS spectrum images were collected with a sample tilt of ∼14° using Gatan DigitalMicrograph 2.32 software, and the files were exported in DM4 format for analysis using Gatan DigitalMicrograph 3.43. Samples for heating experiments were dropcast on Protochips E-chips with carbon support film, and heating experiments were performed using the Protochips Fusion software. OriginPro 2022 was used for spectra visualization as well as peak integration. NIST DTSA-II software was used to generate Monte Carlo simulations of EDS spectra for a bulk, homogeneous diamond containing 0, 1, 2, and 5 atomic % N. Detector specifications were input into the DTSA-II software to match the Bruker XFlash detector used to collect experimental data: windowless silicon drift detector with 0.70 srad solid angle and 0.124 eV FWHM at Mn Kα. In addition to diamond density of 3.51 g/cm^3^, models were generated at 1.5 and 2.0 g/cm^3^ to account for the low-density amorphous carbon on the meteoritic nanodiamond surface, however no appreciable difference was seen in the spectra. CrystalMaker X version 10.8.1 was used to generate graphics for Figure 7.

Synthetic nanodiamonds were provided by FND Biotech, Inc. The nanodiamonds are suspended in water with a concentration of 1 mg/mL. The nanodiamonds contain approximately 10 ppm of the NV centers.^19^

## Supporting Information Available

Two additional examples of NV centers identified through coordinated STEM-EELS/EDS, simulated EDS spectra, and a wide field of view EDS spectrum from a cluster of synthetic nanodiamonds (PDF)

## Abbreviations

NV: nitrogen-vacancy
SPE: single photon emitter
STEM: scanning transmission electron microscope
EELS: electron energy loss spectroscopy
EDS: energy dispersive X-ray spectroscopy
SI: spectrum images
MAADF: medium angle annular dark-field
HAADF: high angle annular dark-field.
