# Supplementary material for: Atomically Precise Detection and Manipulation of Nitrogen-Vacancy Centers in Nanodiamonds
Source: ACS Nano. 2023 Apr 7;17(8):7241–9. doi: 10.1021/acsnano.2c10122 (PMC10134494; doi:10.1021/acsnano.2c10122)
Supplement: Supplementary file 1 — nn2c10122_si_001.pdf [file nn2c10122_si_001.pdf]

Supporting Information

Atomically-precise detection and manipulation of nitrogen-vacancy centers in nanodiamond

Bethany M. Hudak and Rhonda M. Stroud†*

Materials Science and Technology Division, U.S. Naval Research Laboratory, Washington, DC 20375, USA

*bethany.hudak@nrl.navy.mil

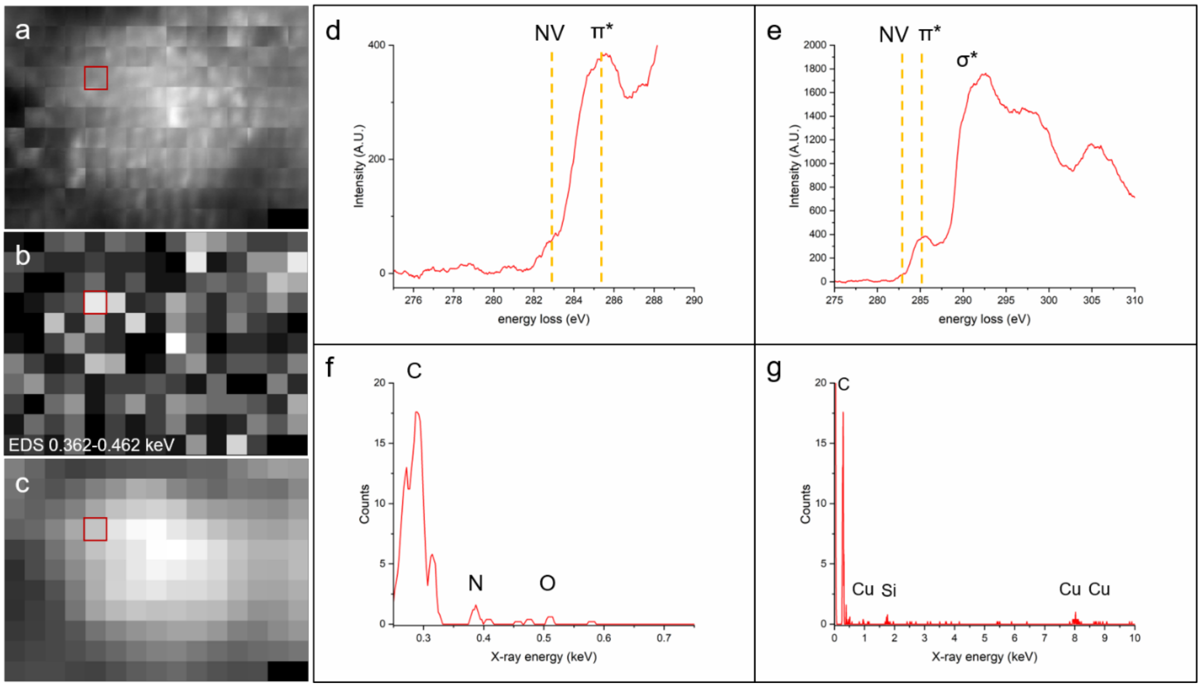

Figure S1. Identification of an NV center in a meteoritic diamond. (a) HAADF image. (b) EDS SI map displaying intensity from 0.362–0.462 keV. (c) EELS SI map. (d) EEL spectrum from red pixel in panel c zoomed in to highlight the peak at 282.4 eV, indicating the presence of an NV center. (e) Full EEL spectrum showing the diamond σ* peak. (f) EDS spectrum from red pixel in panel b zoomed in to show C and N peaks. An O peak from surface contaminants is also present. (g) Full EDS spectrum with Cu system peak and Si contaminant peak present. Si contamination originates from adventitious C from ambient laboratory conditions as well as amorphous C from the acid dissolution process to extract nanodiamonds from the Si-rich meteorite.

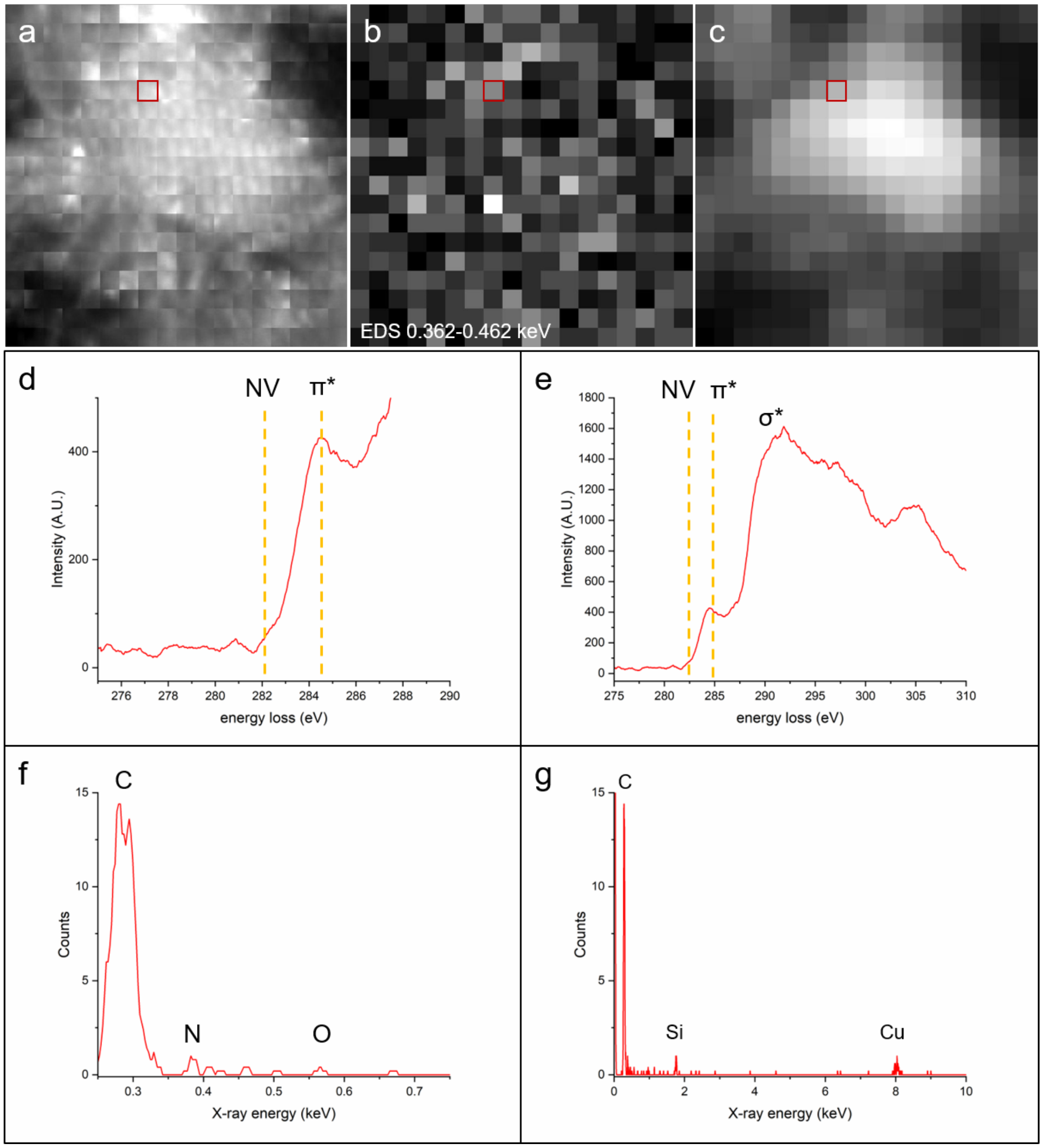

Figure S2. Identification of an NV center in a meteoritic diamond. (a) HAADF image. (b) EDS SI map displaying intensity from 0.362–0.462 keV. (c) EELS SI map. (d) EEL spectrum from red pixel in panel c zoomed in to highlight the peak at 282.4 eV, indicating the presence of an NV center. (e) Full EEL spectrum showing the diamond σ* peak. (f) EDS spectrum from red pixel in panel b zoomed in to show C and N peaks. An O peak from surface contaminants is also present. (g) Full EDS spectrum with Cu system peak and Si contaminant peak present. Si contamination originates from adventitious C from ambient laboratory conditions as well as

amorphous C from the acid dissolution process to extract nanodiamonds from the Si-rich meteorite.

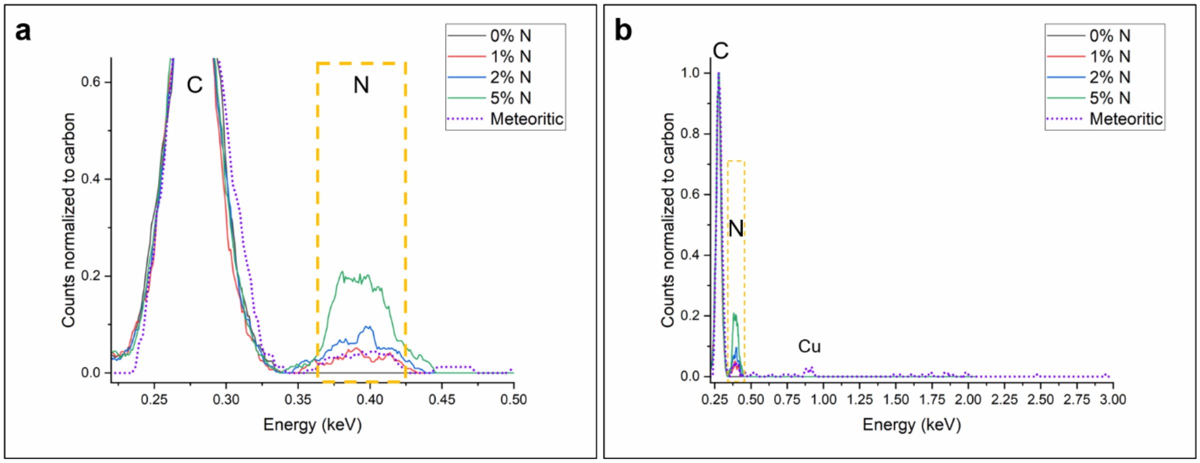

Figure S3. EDS simulation using NIST DTSA-II software. A Monte Carlo model of a 100 nm wide x 5 nm tall block of N-doped diamond was used to generate spectra with 0, 1, 2, and 5 atomic % N. Detector settings were input to match the Bruker XFlash detector used to collect experimental data: windowless silicon drift detector with 0.70 srad solid angle and 0.124 eV FWHM at Mn Kα. In addition to diamond density of 3.51 g/cm^3, models were generated at 1.5 and 2.0 g/cm^3 to account for the low-density amorphous carbon on the meteoritic nanodiamond surface, however no appreciable difference was seen in the spectra. EDS data from the meteoritic nanodiamond NV center in Figure 2 is included to compare to the simulated spectra and matches well with the 1% doping level. (a) EDS spectrum cropped to highlight the N peak at 0.392 eV. The spectra have been smoothed by 10 point adjacent-pixel averaging in Origin Pro 2022 and normalized to the C peak. (b) Wider view of the spectrum displaying the N peak compared to C.

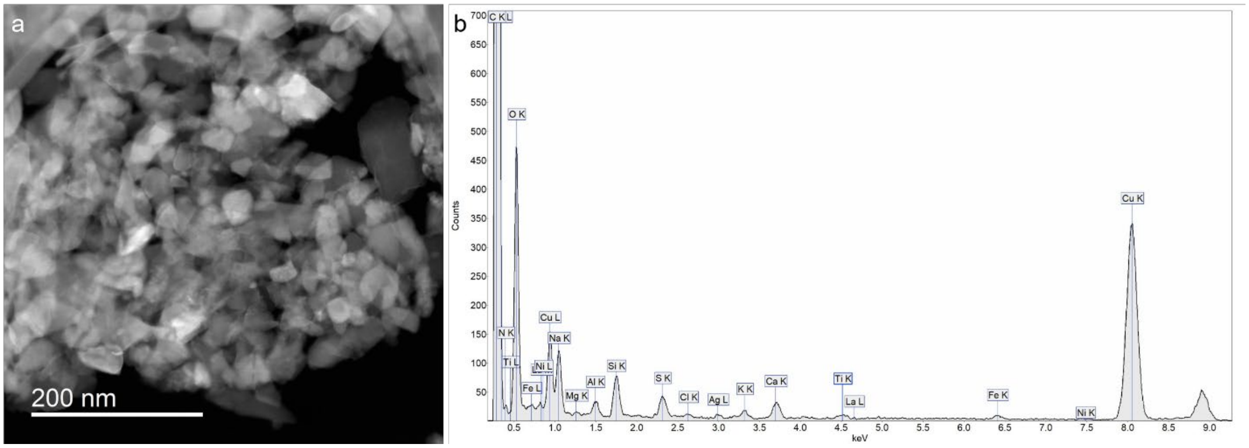

Figure S4. Wide field EDS summed spectrum of synthetic nanodiamonds. (a) MAADF image of a cluster of synthetic nanodiamonds. (b) EDS spectrum acquired over 300 sec from area in (a). The EDS shows a number of impurities present in the sample.
